# Nursing Student Perceptions of Reflective Journaling: A Conjoint Value Analysis

**DOI:** 10.5402/2012/317372

**Published:** 2012-09-05

**Authors:** Thomas J. Hendrix, Maureen O'Malley, Catherine Sullivan, Bernice Carmon

**Affiliations:** School of Nursing, University of Alaska Anchorage, Anchorage, AK 99508, USA

## Abstract

This study used a statistical technique, conjoint value analysis, to determine student perceptions related to the importance of predetermined reflective journaling attributes. An expert Delphi panel determined these attributes and integrated them into a survey which presented students with multiple journaling experiences from which they had to choose. After obtaining IRB approval, a convenience sample of 66 baccalaureate nursing students completed the survey. The relative importance of the attributes varied from a low of 16.75% (format) to a high of 23.58% (time). The model explained 77% of the variability of student journaling preferences (*r* ^2^ = 0.77). Students preferred shorter time, complete confidentiality, one-time complete feedback, semistructured format, and behavior recognition. Students with more experience had a much greater preference for a free-form format (*P* < .05) when compared to students with less journaling experience. Additionally, the results of English as a second language students were significantly different from the rest of the sample. In order to better serve them, educators must consider the relative importance of these attributes when developing journaling experiences for their students.

## 1. Introduction

Reflection is growing in importance as a means to promote a learner-centered environment where the learner is encouraged to learn through the practice of nursing with subsequent reflection. When students are in uncertain new situations, they are expected to use focused thought to apply learned principles to clinical situations and critically evaluate their performance and decisions, rather than focusing on technical knowledge in isolation [1]. The National League for Nursing [2] has reiterated the importance of both reflective and critical thinking through their designation as core competencies for nurse educators. Clinical journals are one of the strategies used throughout the undergraduate nursing curricula to facilitate and guide students' reflective thinking processes.

Reflective journals are considered by (most) faculty members to be essential in fostering an understanding of course concepts and application of concepts to clinical practice; however, students continually question their worth in their anecdotal comments and in formal student course evaluations. Faculty members also voice frustration with insufficient student reflection. Is student dissatisfaction based on reflective journaling in general, the format within an individual course or modality, or some other characteristic of the individual student? It is possible that reflective journals are not consistently reaching the anticipated critical reflection/thinking outcomes that are desired for all students.

This study measured one component of reflective journaling, student perceptions. The purposes of the study were to determine (a) the critical attributes of a reflective journal and relative levels of each attribute, (b) the relative importance of each attribute as perceived by the students, and (c) the perception of relative importance for student subgroups.

## 2. Review of Literature

The reflective learning process for one entering a profession, particularly one entering the health care field, is a very personal experience. The day-to-day experiences in the clinical setting often challenge strongly held beliefs. Personal coping skills are taxed and hopefully modified. Students are expected to consider learned material and make informed decisions. Educators rightfully focus on developing learning tools that include reflection on learned course content, on clinical experiences, on personal values and on their role as the novice nurse.

 At the foundation of the journaling process is the concept of reflection. Reflection, via journal writing, has been an integral component of clinical learning for many years. Reflective journaling is one of the most common methods used to augment clinical learning. Most educators agree that reflective journaling is valuable and contributes to learning [3]; however, they do not agree on how best to structure the reflective journal and if the format of the journal should vary for different student subgroups.

There are several aspects of clinical journaling to consider when studying the student perspective. First, journaling can be viewed as a transformative learning tool for the nursing instructor, challenging the attitudes and the practices of the learner [4]. Educators strive to awaken the novice to the perspective of the client, the family, the role of evidence, or the impact of the multifaceted health care system. Students are encouraged to share the thoughts surrounding their actions and focus on the meaning of their experiences. The focus moves from accomplishing a list of behaviors correctly to the process of learning [5]. Through learning, the learner becomes a new, transformed person.

Secondly, journaling can also be viewed as a skill requiring reasoning and writing expertise. With guidance and support, the clinical reasoning skills seen in the reflective journal can be further developed. In the Epp [3] review of the literature, student writing and reflection became more effective with guidance and the passage of time. Tanner's [6] Clinical Judgment Model lends structure to the clinical reasoning process of the nurse and the nursing student. Reflection is guided using a consistent method, which is designed to promote competence of the baccalaureate nursing student. Clinical reasoning is viewed as a skill, that is, structured, practiced, and ultimately greater proficiency is achieved.

Another aspect to consider is adding structure to the reflective journal, which is thought to promote reflection in nursing students. In an effort to promote learning and promote proficient reflection, nurse educators have developed models [7] or guidelines [8] to assist students to analyze their clinical experiences. Guided reflection has been shown to move students past simply describing events toward more thorough introspection and concept synthesis regarding clinical experiences [9]. By directing students to collect pertinent information, by probing and asking pointed questions, educators direct students toward thinking exercises that foster a deeper understanding. The process of reflection is guided rather than left open to free-form, unstructured comments.

Finally, the reflective journal should be a secure place that encourages the student to question and to elaborate on emotional responses or challenges to existing beliefs. The learning environment is influenced highly by the nature of instructor feedback. Fink [10] defined optimal instructor feedback as being timely, caring, and discriminant. The Lasater [11] Clinical Judgment Rubric describes the components of clinical decision making in a tool that structures the evaluative feedback from an instructor. Feedback is recognized as a key component of a student's reflective journaling experience.

As online educational systems develop, instructors have begun to use group discussion boards to postreflective journals to promote interactive group learning. There is a paucity of research on the use of online group discussion boards for reflective journaling. Students collaborating on journals have demonstrated higher levels of reflection, suggesting benefit from a group process [12, 13]. On the other hand, Craft [9] pointed out that students voiced concerns about the confidentiality of their reflections. The online setting must evoke trust in all members of the group. Collaborative reflective journals have shown promise for some students and present a barrier to others as the journal requires expressing personal thoughts and receiving feedback in a setting that is not private.

The research has shown that many students value reflective journals, but that is not consistent for all students. In one study [14], a group of students indicated that they were skeptical of the benefits of reflective journaling, the act of reflecting did not improve their practice, yet they recognized that there were positive impacts on practice for students who successfully reflected upon their practice. The students were inconsistent regarding their views on reflective journaling. There have been no studies of student perception of the format of reflective journal or studies of student factors that may facilitate or hinder journaling effectiveness. No study has systematically analyzed the perceptions of students related to learning through reflective journals. This study begins to fill that void.

### 2.1. Research Questions

1. What is the relative importance of each attribute involved in the journaling experience?
2. What are changes that would increase satisfaction of students with the journaling experience?
3. Do differences in student characteristics lead to changes in their preferences?

### 2.2. Sample

After receiving IRB approval from University X, students in three undergraduate nursing classes were given the opportunity to voluntarily complete a confidential survey without an instructor present. The survey was offered to second semester junior students (*N* = 41), first semester senior students (*N* = 40), and final semester senior students (*N* = 38). Within this convenience sample, 13, 24, and 19 completed surveys, respectively, were received from these classes for a response rate of 55.5%. There were no missing items on the returned surveys.

### 2.3. Survey Tool

A “Delphi” or expert panel of nurse educators convened to try and determine which attributes of reflective journaling would be viewed by students as the most important characteristics of the journaling process. The panel was comprised of four Doctoral, and two Masters prepared faculty members with 86 cumulative years of experience educating nursing students in the clinical setting. All panel members had used reflective journaling extensively. After extensive discussion and consideration of many possible components of reflective journaling, the panel determined that there were five major attributes contained within the student experience of reflective journaling. After determining these attributes, the panel determined three value levels within each of the attributes. The attributes and levels are described in Table 1.

Additionally, we asked four demographic questions to see if there were relationships between these characteristics and the preferences expressed for the five journaling attributes. These demographic variables are experience, sex, grade point average (GPA), and primary language (ESL). Sex is either male (M) or female (F). GPA is divided into greater than 3.49 (GPA 1), between 2.5 and 3.5 (GPA 2), and less than 2.5 (GPA 3). ESL is a self-reporting variable specifying whether English is their primary language, and students answer either yes (ESLY) or no (ESLN). Experience relates to the number of classes they have already completed where journaling was a component of the coursework. The students were either three trimesters from graduation (Exp 1), two trimesters from graduation (Exp 2), or in the final trimester before graduation (Exp 3). In other words, the more coursework each student had completed, the greater exposure they had had to reflective journaling, as each trimester contains a journaling component in at least one course.

### 2.4. Model

Conjoint analysis is a market-based research analysis model where potential and/or actual customers make choices based on their preferences. These choices are based on the individual attributes of the product under consideration and are considered jointly (conjoint). For example, all else equal, most consumers would choose the cheapest computer. Similarly, all else equal, most consumers would choose the fastest computer. However, they cannot have both of these attributes simultaneously because customers are forced to pay additional money for the faster computer should they desire more speed. In fact, all else is rarely equal. People make tradeoffs every day as they try and balance the partial worth of many attributes like speed, color, size, power, convenience, durability, prestige, necessity, and price (among others). A similar model exists in education. Students, like computer purchasers, have preferences about what is important to them and are, in effect, buyers of nursing education. Like the manufacturers of computers who care greatly about the preferences of their customers, the “sellers” of nursing education should also care about the preferences of their customers. For example, in reflective journaling, students might desire a short time to complete their journaling requirement and, at the same time, want a transformative experience which requires more time. Therefore, like the computer shopper, nursing students make choices that involve tradeoffs.

### 2.5. Methods

Each attribute and its corresponding levels were entered into the conjoint value analysis (CVA) software program which automatically generates a survey. The survey began with the four demographic questions previously described and followed with twenty pair-wise discrete choice questions. Each question presented the student respondent with a choice of two journaling experiences from which they had to choose one. Respondents were free to choose along a nine-point Likert scale from “strongly prefer left” (scale 1) to “somewhat prefer left” (scale 3) to “indifferent” (scale 5) to “somewhat prefer right” (scale 7) to “strongly prefer right” (scale 9). Each attribute is present in all choices, but the levels within each attribute are varied, and these variations are shuffled with each succeeding question. Additionally, the CVA questionnaire generator automatically moves attribute levels from left to right as the respondent proceeds through the questionnaire. A typical conjoint question is in Table 2, and the complete survey is available upon request.

After data entry, the CVA software runs multiple ordinary least squares (OLSs) functions by performing simultaneous regressions of the five independent attribute variables on the dependent variable of preference. For a detailed explanation of CVA, please refer to the Sawtooth Software Technical Paper Series referenced in the bibliography. The results are presented as average utilities (preferences), and the average importance of each attribute is estimated, and an *R* ^2^ is calculated. The results are then exported to the Sawtooth Marketing Simulation (SMRT) software which runs a series of simulation algorithms where the different levels of the same attribute are compared to each other while holding all other attributes the same. These results estimate the share preferences for each attribute level relative to other levels. Through multiple iterations, all preferences for each level are estimated in relationship to each other by simply dividing one into the next. Finally, to estimate the changes (if any) that the demographic variables have on the preferences of attributes levels within reflective journaling, the Marketing Simulation software takes the attribute preference data and filters it through the four demographic questions and provides a result containing product shares of preference and standard errors. Through these two statistics, confidence intervals and *P* values (*Z* scores) are then calculated.

### 2.6. Results and Analysis

The results of CVA OLS are presented in Tables 3 and 4. The importance (Table 3) of the five attributes varies from a low of 16.75% (format) to a high of 23.58% (time). The lack of any one attribute being extremely high or low relative to the other attributes suggests that all five attributes were considered and paid attention to by the respondents. In other words, all attributes were relatively important.

This is further reinforced by an *R* squared (see Table 4) of 0.777. That is, this model explains 77.7% of the variability of journaling preferences among nursing students and is clearly superior to a random predictor of this variation. The utilities (see Table 4) are interval-scaled values without a real zero. Therefore, utilities represent weights relative to each other, and the sum of the three levels equals zero.

It is clear that students prefer a shorter time, complete confidentiality, recognition of their behavior, one-time complete feedback, and a semistructured format. However, because these are interval values, they provide preferences only. For these data to be useful in the real world of developing courses and, more specifically, journaling experiences, there must be a real zero. Additionally, to accurately compare different levels of any single attribute, all other attributes must be held constant. The SMRT uses a randomized first choice simulation algorithm to accomplish this. For a detailed explanation of the SMRT software package, please refer to the Sawtooth research paper on market simulations referenced in the bibliography.  Table 5 presents these results with subsequent calculations.

Students prefer a weekly time commitment of 15 minutes over 30 minutes (*P* < 0.05), and they prefer 30 minutes over 45 minutes (*P* < 0.05). By decreasing the time commitment from 45 to 30 minutes, student satisfaction increases by 36%. By decreasing time from 30 to 15 minutes, student satisfaction increases by 15% and if students are currently spending 45 minutes a week and an instructor reduces that to 15 minutes (*P* < 0.05), student satisfaction increases by 54%. 

Students require anonymous journals. Both iterations that contained the “open” (nonanonymous) option were rejected by students. By moving the journaling experience from open to either completely confidential (*P* < 0.05) or to shared content (*P* < 0.05), student satisfaction increases by 36% and 25%, respectively. However, it is less clear whether students have a problem sharing their thoughts anonymously. The tails of the two 95% C.I. overlap so the *P* value is clearly greater than 0.05. However, by taking the square root of the squared sum of the two separate standard error terms and dividing that into the difference of the partial shares, a *P* value is easily found in a *Z* table (0.07). Therefore, students also prefer complete confidentiality to anonymous sharing (*P* = 0.07), but their satisfaction only increases by 9% by moving from the latter to the former.

Students want results from their journaling efforts though they are relatively indifferent whether they simply learn to recognize their behaviors or have those behaviors transformed. In both iterations where “no result” was an option, students rejected it. In fact, by changing the journaling experience from one where the students perceive that no change has occurred to one where they either recognize their behaviors (*P* < 0.05) or have them transformed (*P* < 0.05), student satisfaction increases by 41% and 38%, respectively. However, the two 95% C.I. that compared recognition to transformation almost completely overlap. There is no perceivable difference. So although instructors may think that it is a higher level of learning for students to be transformed versus simply recognizing positive and negative behaviors, students do not value that difference. 

Students value feedback over simply checking their work for completeness although they are indifferent whether that feedback comes only once or multiple times. By moving from a model of checking for completeness to one where students receive feedback once (*P* < 0.05), student satisfaction increases by 11%. However, the 95% intervals for checking for completeness versus multiple feedbacks have overlapping tails and the *P* value (0.06) is calculated (as above) using the *Z* table. Finally, students are indifferent to a single feedback or multiple feedbacks. The 95% C.I. for both of these levels almost completely overlap, and there is no difference.

Students do not value a completely structured format. By moving from a completely structured format to either a semi-structured (*P* < 0.05) or open format (*P* = 0.06) student satisfaction increases by 10% and 9%, respectively. However, students did not perceive much difference between the semi-structured or open format. There was no difference.

Finally, to estimate the changes (if any) that the demographic variables have on the preferences of attributes levels within reflective journaling, the attribute preference data was filtered through the four demographic questions. The significant results are presented in Table 6. Most of the demographic categories had no significant effect on student preferences, and these are not reported though the data is available upon request. 

The sex and GPA of the respondents had no significant effect on student preferences for journaling attributes. That is, all calculated confidence intervals that compared these two demographic variables had a great deal of overlap. Thus, they were considered the same. The experience level of the students did have an effect on the value of time and the preference of format. Those students with less experience (Exp 1) had a much greater preference for less time spent journaling (*P* < 0.023) and a much greater preference for a structured format (*P* < 0.39). Conversely, those students with the most experience (Exp 3) had a much greater preference for a free-form format (*P* < 0.39) when compared to students with less journaling experience.

Students that self-reported English as their second language (ESLY) viewed the results of journaling differently. That is, although the “no effect” level of result was perceived negatively by all students, it was perceived much more negatively by non-ESL students. Additionally, instructor feedback had a lower perceived value among ESL students. Those students whose first language was English (ESLN) preferred feedback to no feedback, and ESL students did not. The results for the ESL demographic should be viewed with some caution as there were only 2 students reporting themselves as ESL. Although the weakness of a small cohort is captured in the standard error calculation, which leads directly to the *P* value, it still gives pause. We report those results here because mathematically the differences were significant but will leave it up to the reader to decide.

### 2.7. Answers to Research Questions

*Question *1*: what is the relative importance of each of the five attributes involved in the journaling experience?* In descending order, the most important characteristics of journaling are time, confidentiality, results, feedback and format (Table 1).

*Question *2*: what are the changes that would most increase the satisfaction of students with the journaling experience?* To increase satisfaction for students, instructors should strive to reduce the time students spend journaling, maintain anonymity (though content might be shared), and instill a perception in students that reflective journaling will have a tangible effect on each student's ability to transform or simply recognize their behaviors (Table 5, far right column).

*Question *3*: do differences in student characteristics lead to changes in their preferences about the journaling experience.* There were no differences noted based on sex or academic achievement. However, students with less experience in journaling value time more than the entire cohort and students with more experience prefer an open format with fewer instructor cues. Additionally, ESL students placed less value on instructor feedback and behavior recognition or transformation. However, because of the small sample size, these results should be viewed with some caution (Table 6).

## 3. Discussion

Students strongly value their time. This is the one dimension of the reflective journal that has not been studied. The Delphi panel included this dimension as an attribute of the journal recognizing that it is a paramount concern of nursing students. These students clearly indicated a preference for journaling exercises that required less time. Nursing instructors will probably read this and recognize the challenge of creating journaling exercises that are both time efficient and produce the requisite level of reflection. However, students would highly value new journaling exercises that prove to be more time efficient.

Students value anonymity in journaling. This is a concern as online discussion systems become more widely used. Online, discussion systems should be developed that include areas for confidential discussion and individual feedback, as well as open discussions areas where students can benefit from interaction and collaboration. In the age of the electronic classroom, anonymity and collaboration must be balanced.

It is interesting to note the undergraduate student's comments on the structured journal considering the development of various models or guidelines [7, 8]. Students preferred either a semi-structured or an open, free-form journal. They do not value a completely structured journal. Educators working on models that structure student responses should be advised to insure that exercises include areas for students to write freely without predetermined structure. There is evidence that using a model or structured journal early in a nursing program and moving to a predominantly unstructured journal later in the program will be valued more highly by students. Instructors should insure that whether using a model or not, students value substantive feedback after they have prepared reflective journals.

ESL students viewed journaling differently compared to non-ESL students. C. Kuo [15] developed an online writing system to support nonnative students with writing exercises. The system offers a supportive environment encouraging writing practice, peer review, and use of an e-portfolio, online dictionary, phrase lists, and so forth. The ESL student practices writing and benefits from peer review and revision. ESL students may face unique challenges in completing reflective journaling assignments. Research findings are beginning to reveal more about the kinds of support that will benefit this group of students. 

### 3.1. Limitations

The R squared for this study was 0.777. This is quite robust and explains 77.7% of the variation within the journaling model. However, 23.3% of the variability was not explained by this model. Therefore, there exists a risk that a significant variable was overlooked by the Delphi panel and not included in the survey. The omission of significant variables can lead to specification error which has the potential to bias coefficients and results.

The students in our sample attend the same school, are taught by the same teachers, and live in the state of X. Journaling experiences nationwide or worldwide might be quite different than those experienced by the students at our institution and, therefore, these results might not generalize to other populations in other institutions across the country.

When accomplishing crosstabs with the demographic variables, there were some very small subgroups. For example, there were only 2 ESL students, 6 male students, and 3 who reported a GPA under 2.5. All calculations in this study make use of the standard error term, and standard errors take into account small sample sizes. However, we would still view all of the small sample conclusions with some caution. Similarly, regardless of sample size, a *P* value of 0.05 still means that we are only correct 19 out of 20 times and, therefore, could be wrong.

## 4. Conclusions

Marketing professionals have surveyed their customers and potential customers in an effort to provide the product or service that is most desired by them. We also wanted to find out what aspects of journaling our customers (students) value. This study applied the statistical method of conjoint value analysis, a sound quantitative modeling method that has been used in the field of marketing for many years. Results indicated that all students value time, confidentiality, results, feedback, and format, in that order. The less-experienced students valued a structured journal, while the more advanced students valued a more free-form journal. Nurse educators should consider these attributes when developing and refining the journals in their courses. While journaling is a process that supports learning, it is also very personal. Students must see that the journaling system is confidential and that they will receive thoughtful feedback from the instructors. This study helps us better understand nursing students, our customers, and to better design journaling experiences that future students might find more satisfying. More research is needed to test new journaling systems as well as to explore the unique needs of student subgroups, particularly ESL students.

## Supplementary Material

There are four brief information questions at the top followed by 20 questions about journalling. Thi survey is completely confidential and the information that you provide will be analyzed and will probably result in changes in the way that we require journalling to be accomplished at the School of Nursing.

## Figures and Tables

**Table 1:** Operational definitions of attributes and levels.

Attribute	Definition	Level 1	Level 2	Level 3
Time	The time (in minutes) it takes for the student to complete their weekly journaling requirement.	15 minutes	30 minutes	45 minutes
Result	The desired impact on the self-perceived behavior of the student	No impact on student behavior	Recognize positive and negative behaviors	Transforms student behavior
Feedback	The amount of feedback that the instructor provides to the student	Checks for completeness only	Provides one-time comprehensive feedback	Provides “give and take” with multiple feedback opportunities.
Structure	The journaling format that the students must follow	Structured questions	Combination of structure and free-flow	Free-flow open forum
Confidentiality	The presence or lack of confidentiality of the author and/or content of reflective journaling	Name and content are completely confidential	Content can be shared but name remains confidential	No confidentiality of either name or content

**Table 2:** A typical conjoint question.

If you had to choose one of these two descriptions of reflective journaling for an upcoming class, which would you choose?								
Takes 45 minutes to complete					Takes 15 minutes to complete			
Result helps me recognize my positive and negative behaviors					Result has no effect on my behavior			
Instructor checks for completeness and provides one-time feedback					Instructor checks for completeness and provides multiple back and forth feedback			
Student comments are shared anonymously with other students					Student journal is completely confidential and not shared with other students			
Student writing responds to structured instructor questions					Student writing is semistructured with open-ended and structured questions.			

o	o	o	O	o	o	o	o	o

Strongly prefer left		Somewhat prefer left		Indifferent		Somewhat prefer right		Strongly prefer right

**Table 3:** Attribute importance (total = 100%).

Attribute	Importance (%)
Time	23.58
Confidentiality	21.30
Result	20.78
Feedback	17.58
Format	16.75

**Table 4:** CVA OLS average utilities (zero-centered differentials).

Attribute	Level	Utility	Attribute	Level	Utility
	15 minutes	35.48		No effect	−42.08
Time	30 minutes	10.31	Result	Recognize behavior	24.27
	45 minutes	−45.79		Transform behavior	17.81

	Complete	24.80		Check only	−14.23
Confidentiality	Content only	10.04	Feedback	One time	9.26
	None	−34.84		Multiple	4.98

				Free flow	3.26
			Format	Semi structured	6.03
				Structured	−9.29
			*R* squared	0.777	

**Table 5:** Partial shares of preference.

Run	Level	Share of preference	Standard error	Lower (95%) C.I.	Upper (95%) C.I.	Statistically different	Satisfaction ratio (Hi share/Lo share)	Potential increase in satisfaction
Time 1	15 min	53.36	1.15	51.11	55.61	Yes	1.15	15% (*P* < 0.05)
	30 min	46.64		44.39	48.90			
Time 2	30 min	57.68	1.06	55.6	59.76	Yes	1.36	36% (*P* < 0.05)
	45 min	42.32		40.24	44.4			
Time 3	15 min	60.59	1.52	57.61	63.57	Yes	1.54	54% (*P* < 0.05)
	45 min	39.41		36.43	42.39			

Conf 1	Complete	52.12	1.10	49.96	54.28	Tails overlap	1.09	9% (*P* = 0.07)
	Content	47.88		45.72	50.04			
Conf 2	Content	55.53	1.41	52.77	58.29	Yes	1.25	25% (*P* < 0.05)
	Open	44.47		41.71	47.23			
Conf 3	Complete	57.64	1.50	54.7	60.58	Yes	1.36	36% (*P* < 0.05)
	Open	42.36		39.42	45.3			

Result 1	None	41.47	1.27	38.98	43.96	Yes	1.41	41% (*P* < 0.05)
	Recognize	58.53		56.04	61.02			
Result 2	Recognize	50.59	1.06	48.51	52.67	No	1.02	2% Nonsignificant
	Transform	49.41		47.33	51.49			
Result 3	None	41.96	1.11	39.78	44.14	Yes	1.38	38% (*P* < 0.05)
	Transform	58.04		55.86	60.22			

Fdbk 1	Check	47.49	1.03	45.4	49.58	Yes	1.11	11% (*P* < 0.05)
	One time	52.51		50.42	54.6			
Fdbk 2	One time	50.05	1.11	47.87	52.23	No	1.002	0.2% Nonsignificant
	Multiple	49.95		47.77	52.13			
Fdbk 3	Check	47.54	1.27	45.05	50.03	Tails overlap	1.10	10% (*P* = 0.06)
	Multiple	52.46		49.97	54.95			

Format 1	Free flow	50.08	1.23	47.67	52.49	No	1.003	0.3% Nonsignificant
	Semi	49.92		47.51	52.33			
Format 2	Semi	52.46	1.07	50.36	54.56	Yes	1.10	10% (*P* < 0.05)
	Structured	47.54		45.44	49.64			
Format 3	Free flow	52.12	1.59	49	55.24	Tails overlap	1.09	9% (*P* = 0.06)
	Structured	47.88		44.76	51			

**Table 6:** Demographic effect on preference shares.

Attribute	Demographic share				Standard error		Calc *Z *	*P* value	Different
Time	Exp 1 *N* = 13	Exp 2 *N* = 34	Exp 3 *N* = 16						
15 min	67.01	59.37	58.38	3.03	1.48	3.84	−2.26	<0.023	Yes
45 min	32.99	40.63	41.62						

Format									
Free flow	44.80	51.43	58.35	3.56	2.05	2.66	−2.06	<0.39	Yes
Structured	55.20	48.57	41.65						

Result	ESLN *N* = 64	ESYN *N* = 2							
No effect	41.36	44.89		1.31	1.32		−1.86	<0.57	Yes
Recognize	58.64	55.11							

Feedback									
Check only	47.34	52.26		1.05	1.89		−2.27	<0.02	Yes
One time	52.66	47.74							
