# Supplementary material for: Nursing Student Perceptions of Reflective Journaling: A Conjoint Value Analysis
Source: ISRN Nurs. 2012 Sep 5;2012:317372. doi: 10.5402/2012/317372 (PMC3440922; doi:10.5402/2012/317372)
Supplement: Supplementary file 1 — There are four brief information questions at the top followed by 20 questions about journalling. Thi survey is completely confidential and the information that you provide will be analyzed and will probably result in changes in the way that we require journalling to be accomplished at the School of Nursing. [file 317372.f1.pdf]

School of Nursing Survey of Journalling Preferences

Welcome to our survey! We are interested in your thoughts about refelctive journals. There are 5 attributes of reflective journals that we will examine today. Here is a brief explaination of these attributes.

1. Time: This is the amount of time it takes you to complete your journalling requirement each week. Time is listed in minutes.

2. Growth: This is your perception of the learning that takes place as a result of your efforts in the journalling process. Your choices are either (a) no effect; (b) you recognize your behaviors; (c) your behaviors are transformed

3. Confidentiality: This is the degree that you are comfortable with the instructor sharing your name and/or comments with the class. Your choices are: (a) Strict confidentiality; (b) Anonymous sharing where your thoughts are shared but not your name; (c) Completely open without any confidentiality.

4. Instructor Feedback: This is the quality and quantity of feedback you receive from your instructor. Any of your choices contain the underlying assumption that you receive credit for your work. Your choices are: (a) Instructor puts a simple check mark on your work; (b) Instructor provides detailed feedback but only once; (c) Instructor provides specific feedback and allows you the opportunity to respond to that feedback and then additional feedback may ensue.

5. Format: This is the structure of the journal. Your choices are: (a) Open format. That is, there are no instructor questions for you to answer; (b) Semi-structured: This is a combination of structured questions and open questions; (c) Instructor-provided structured questions

This Survey: You will be presented two choices of journals and will be asked which you prefer. There will be a preference scale below each question. Go ahead and choose the one that contains the most qualities you prefer even if neither is ideal. You also have the opportunity to select the degree to which you prefer it. If you choose a "bubble" all of the way to the left, you are saying that the choice on the left side is greatly preferred then the one on the right. If you choose the far right, you are telling us that you greatly prefer the far right. The "bubbles" between the two extremes provide you the opportunity to select less than a "greatly preferred" choice. As you progress through the survey your choices will constantly change. Continue to choose the best fit for you even if neither is perfect.

There are four brief information questions at the top followed by 20 questions about journalling. Thi survey is completely confidential and the information that you provide will be analyzed and will probably result in changes in the way that we require journalling to be accomplished at the School of Nursing. Thank you!

1. In which class are you currently enrolled?

- a) NS313/315
- b) NS401/406
- c) NS411/416

2. What is your gender?

- a) Male
- b) Female

3. What is your current cumulative grade point average?

- a) greater than 3.49
- b) greater than 2.99 and less then 3.50
- c) gretaer than 2.49 and less than 3.0
- d) less than 2.50

4. Is English your primary language?

- a) Yes
- b) No

5.

If you had to choose one of these two descriptions of reflective journalling for an upcoming class, which would you choose?

Takes 45 minutes weekly to complete	or	Takes 15 minutes weekly to complete
Result helps me recognizes my positive and negative behaviors		Result has no effect on my behavior
Instructor checks for completeness and provides one time feedback		Instructor checks for completeness and provides multiple back and forth feedback
Students journal comments are shared anonymously with other students		Student journal is completely confidential and not shared with other students
Student writing responds to structured instructor questions		Student writing is semi-structured with open-ended and structured questions

a	b	c	d	e	f	g	h	i
Strongly Prefer Left		Somewhat Prefer Left		Indifferent		Somewhat Prefer Right		Strongly Prefer Right

6.

If you had to choose one of these two descriptions of reflective journalling for an upcoming class, which would you choose?

Takes 30 minutes weekly to complete	or	Takes 15 minutes weekly to complete
Result has no effect on my behavior		Result positively transforms my behavior
Instructor checks for completeness and provides multiple back and forth feedback		Instructor will check for completeness only
Student journal is completely confidential and not shared with other students		Student comments are presented in an open forum with no confidentiality
Student writing is semi-structured with open-ended and structured questions		Student writing is free-flow without instructor cues

a	b	c	d	e	f	g	h	i
Strongly Prefer Left		Somewhat Prefer Left		Indifferent		Somewhat Prefer Right		Strongly Prefer Right

7.

If you had to choose one of these two descriptions of reflective journalling for an upcoming class, which would you choose?

Option Left		Option Right
Takes 15 minutes weekly to complete		Takes 45 minutes weekly to complete
Result helps me recognizes my positive and negative behaviors		Result positively transforms my behavior
Instructor will check for completeness only	or	Instructor checks for completeness and provides one time feedback
Student journal is completely confidential and not shared with other students		Students journal comments are shared anonymously with other students
Student writing responds to structured instructor questions		Student writing is semi-structured with open-ended and structured questions

a	b	c	d	e	f	g	h	i
Strongly Prefer Left		Somewhat Prefer Left		Indifferent		Somewhat Prefer Right		Strongly Prefer Right

8.

If you had to choose one of these two descriptions of reflective journalling for an upcoming class, which would you choose?

Option Left		Option Right
Takes 15 minutes weekly to complete		Takes 45 minutes weekly to complete
Result positively transforms my behavior		Result helps me recognizes my positive and negative behaviors
Instructor checks for completeness and provides one time feedback	or	Instructor will check for completeness only
Students journal comments are shared anonymously with other students		Student comments are presented in an open forum with no confidentiality
Student writing is free-flow without instructor cues		Student writing responds to structured instructor questions

a	b	c	d	e	f	g	h	i
Strongly Prefer Left		Somewhat Prefer Left		Indifferent		Somewhat Prefer Right		Strongly Prefer Right

9.

If you had to choose one of these two descriptions of reflective journalling for an upcoming class, which would you choose?

Option Left		Option Right
Takes 45 minutes weekly to complete		Takes 30 minutes weekly to complete
Result positively transforms my behavior		Result has no effect on my behavior
Instructor checks for completeness and provides multiple back and forth feedback	or	Instructor will check for completeness only
Student comments are presented in an open forum with no confidentiality		Student journal is completely confidential and not shared with other students

Student writing is semi-structured with open-ended and structured questions		Student writing is free-flow without instructor cues

a	b	c	d	e	f	g	h	i
Strongly Prefer Left		Somewhat Prefer Left		Indifferent		Somewhat Prefer Right		Strongly Prefer Right

10.

If you had to choose one of these two descriptions of reflective journalling for an upcoming class, which would you choose?

Takes 15 minutes weekly to complete		Takes 30 minutes weekly to complete
Result positively transforms my behavior		Result has no effect on my behavior
Instructor checks for completeness and provides multiple back and forth feedback	or	Instructor checks for completeness and provides one time feedback
Student journal is completely confidential and not shared with other students		Students journal comments are shared anonymously with other students
Student writing is free-flow without instructor cues		Student writing is semi-structured with open-ended and structured questions

a	b	c	d	e	f	g	h	i
Strongly Prefer Left		Somewhat Prefer Left		Indifferent		Somewhat Prefer Right		Strongly Prefer Right

11.

If you had to choose one of these two descriptions of reflective journalling for an upcoming class, which would you choose?

Takes 30 minutes weekly to complete		Takes 15 minutes weekly to complete
Result has no effect on my behavior		Result helps me recognizes my positive and negative behaviors
Instructor checks for completeness and provides multiple back and forth feedback	or	Instructor checks for completeness and provides one time feedback
Student comments are presented in an open forum with no confidentiality		Student journal is completely confidential and not shared with other students
Student writing is free-flow without instructor cues		Student writing is semi-structured with open-ended and structured questions

a	b	c	d	e	f	g	h	i
Strongly Prefer		Somewhat Prefer Left		Indifferent		Somewhat Prefer		Strongly Prefer

Left						Right		Right

12.

If you had to choose one of these two descriptions of reflective journalling for an upcoming class, which would you choose?

Takes 30 minutes weekly to complete<br>Result has no effect on my behavior<br>Instructor checks for completeness and provides one time feedback<br>Student comments are presented in an open forum with no confidentiality<br>Student writing responds to structured instructor questions	or	Takes 45 minutes weekly to complete<br>Result positively transforms my behavior<br>Instructor will check for completeness only<br>Students journal comments are shared anonymously with other students<br>Student writing is semi-structured with open-ended and structured questions

a	b	c	d	e	f	g	h	i
Strongly Prefer Left		Somewhat Prefer Left		Indifferent		Somewhat Prefer Right		Strongly Prefer Right

13.

If you had to choose one of these two descriptions of reflective journalling for an upcoming class, which would you choose?

Takes 45 minutes weekly to complete<br>Result has no effect on my behavior<br>Instructor checks for completeness and provides one time feedback<br>Student journal is completely confidential and not shared with other students<br>Student writing is free-flow without instructor cues	or	Takes 30 minutes weekly to complete<br>Result helps me recognizes my positive and negative behaviors<br>Instructor checks for completeness and provides multiple back and forth feedback<br>Students journal comments are shared anonymously with other students<br>Student writing is semi-structured with open-ended and structured questions

a	b	c	d	e	f	g	h	i
Strongly Prefer Left		Somewhat Prefer Left		Indifferent		Somewhat Prefer Right		Strongly Prefer Right

14.

If you had to choose one of these two descriptions of reflective journalling for an upcoming class, which would you choose?

Takes 15 minutes weekly to complete<br><br>Result has no effect on my behavior<br><br>Instructor checks for completeness and provides multiple back and forth feedback<br><br>Students journal comments are shared anonymously with other students<br><br>Student writing responds to structured instructor questions	or	Takes 30 minutes weekly to complete<br><br>Result helps me recognizes my positive and negative behaviors<br><br>Instructor checks for completeness and provides one time feedback<br><br>Student journal is completely confidential and not shared with other students<br><br>Student writing is semi-structured with open-ended and structured questions

a	b	c	d	e	f	g	h	i
Strongly Prefer Left		Somewhat Prefer Left		Indifferent		Somewhat Prefer Right		Strongly Prefer Right

15.

If you had to choose one of these two descriptions of reflective journalling for an upcoming class, which would you choose?

Takes 30 minutes weekly to complete<br><br>Result positively transforms my behavior<br><br>Instructor checks for completeness and provides one time feedback<br><br>Student comments are presented in an open forum with no confidentiality<br><br>Student writing is free-flow without instructor cues	or	Takes 15 minutes weekly to complete<br><br>Result has no effect on my behavior<br><br>Instructor checks for completeness and provides multiple back and forth feedback<br><br>Students journal comments are shared anonymously with other students<br><br>Student writing responds to structured instructor questions

a	b	c	d	e	f	g	h	i
Strongly Prefer Left		Somewhat Prefer Left		Indifferent		Somewhat Prefer Right		Strongly Prefer Right

16.

If you had to choose one of these two descriptions of reflective journalling for an upcoming class, which would you choose?

Takes 45 minutes weekly to complete<br><br>Result helps me recognizes my positive and negative behaviors<br><br>Instructor will check for completeness only	or	Takes 30 minutes weekly to complete<br><br>Result positively transforms my behavior<br><br>Instructor checks for completeness and provides multiple back and forth feedback

Students journal comments are shared anonymously with other students<br><br>Student writing is free-flow without instructor cues		Student comments are presented in an open forum with no confidentiality<br><br>Student writing responds to structured instructor questions

a	b	c	d	e	f	g	h	i
Strongly Prefer Left		Somewhat Prefer Left		Indifferent		Somewhat Prefer Right		Strongly Prefer Right

17.

If you had to choose one of these two descriptions of reflective journalling for an upcoming class, which would you choose?

Takes 15 minutes weekly to complete<br><br>Result helps me recognizes my positive and negative behaviors<br><br>Instructor will check for completeness only<br><br>Student comments are presented in an open forum with no confidentiality<br><br>Student writing is semi-structured with open-ended and structured questions	or	Takes 45 minutes weekly to complete<br><br>Result has no effect on my behavior<br><br>Instructor checks for completeness and provides one time feedback<br><br>Student journal is completely confidential and not shared with other students<br><br>Student writing responds to structured instructor questions

a	b	c	d	e	f	g	h	i
Strongly Prefer Left		Somewhat Prefer Left		Indifferent		Somewhat Prefer Right		Strongly Prefer Right

18.

If you had to choose one of these two descriptions of reflective journalling for an upcoming class, which would you choose?

Takes 45 minutes weekly to complete<br><br>Result has no effect on my behavior<br><br>Instructor will check for completeness only<br><br>Student journal is completely confidential and not shared with other students<br><br>Student writing is semi-structured with open-ended and structured questions	or	Takes 15 minutes weekly to complete<br><br>Result helps me recognizes my positive and negative behaviors<br><br>Instructor checks for completeness and provides one time feedback<br><br>Students journal comments are shared anonymously with other students<br><br>Student writing responds to structured instructor questions

a	b	c	d	e	f	g	h	i
Strongly		Somewhat		Indifferent		Somewhat		Strongly

Prefer Left	Prefer Left		Prefer Right	Prefer Right

19.

If you had to choose one of these two descriptions of reflective journalling for an upcoming class, which would you choose?

Left		Right
Takes 15 minutes weekly to complete	or	Takes 45 minutes weekly to complete
Result has no effect on my behavior		Result positively transforms my behavior
Instructor will check for completeness only		Instructor checks for completeness and provides multiple back and forth feedback
Student comments are presented in an open forum with no confidentiality		Student journal is completely confidential and not shared with other students
Student writing is semi-structured with open-ended and structured questions		Student writing is free-flow without instructor cues

a	b	c	d	e	f	g	h	i
Strongly Prefer Left		Somewhat Prefer Left		Indifferent		Somewhat Prefer Right		Strongly Prefer Right

20.

If you had to choose one of these two descriptions of reflective journalling for an upcoming class, which would you choose?

Left		Right
Takes 15 minutes weekly to complete	or	Takes 45 minutes weekly to complete
Result positively transforms my behavior		Result has no effect on my behavior
Instructor checks for completeness and provides one time feedback		Instructor will check for completeness only
Student journal is completely confidential and not shared with other students		Student comments are presented in an open forum with no confidentiality
Student writing is semi-structured with open-ended and structured questions		Student writing is free-flow without instructor cues

a	b	c	d	e	f	g	h	i
Strongly Prefer Left		Somewhat Prefer Left		Indifferent		Somewhat Prefer Right		Strongly Prefer Right

21.

If you had to choose one of these two descriptions of reflective journalling for an upcoming class, which would you choose?

Takes 30 minutes weekly to complete	or	Takes 15 minutes weekly to complete
Result helps me recognizes my positive and negative behaviors		Result has no effect on my behavior
Instructor will check for completeness only		Instructor checks for completeness and provides one time feedback
Student journal is completely confidential and not shared with other students		Student comments are presented in an open forum with no confidentiality
Student writing responds to structured instructor questions		Student writing is semi-structured with open-ended and structured questions

a	b	c	d	e	f	g	h	i
Strongly Prefer Left		Somewhat Prefer Left		Indifferent		Somewhat Prefer Right		Strongly Prefer Right

22.

If you had to choose one of these two descriptions of reflective journalling for an upcoming class, which would you choose?

Takes 30 minutes weekly to complete	or	Takes 15 minutes weekly to complete
Result positively transforms my behavior		Result helps me recognizes my positive and negative behaviors
Instructor will check for completeness only		Instructor checks for completeness and provides multiple back and forth feedback
Students journal comments are shared anonymously with other students		Student journal is completely confidential and not shared with other students
Student writing responds to structured instructor questions		Student writing is free-flow without instructor cues

a	b	c	d	e	f	g	h	i
Strongly Prefer Left		Somewhat Prefer Left		Indifferent		Somewhat Prefer Right		Strongly Prefer Right

23.

If you had to choose one of these two descriptions of reflective journalling for an upcoming class, which would you choose?

Takes 45 minutes weekly to complete	or	Takes 30 minutes weekly to complete
Result helps me recognizes my positive and negative behaviors		Result positively transforms my behavior
Instructor checks for completeness and provides one time feedback		Instructor will check for completeness only
Student comments are presented in an		Students journal comments are shared

open forum with no confidentiality<br><br>Student writing responds to structured instructor questions	anonymously with other students<br><br>Student writing is free-flow without instructor cues

a	b	c	d	e	f	g	h	i
Strongly Prefer Left		Somewhat Prefer Left		Indifferent		Somewhat Prefer Right		Strongly Prefer Right

24.

If you had to choose one of these two descriptions of reflective journalling for an upcoming class, which would you choose?

Takes 30 minutes weekly to complete<br><br>Result helps me recognizes my positive and negative behaviors<br><br>Instructor checks for completeness and provides multiple back and forth feedback<br><br>Students journal comments are shared anonymously with other students<br><br>Student writing is free-flow without instructor cues	or	Takes 15 minutes weekly to complete<br><br>Result positively transforms my behavior<br><br>Instructor will check for completeness only<br><br>Student journal is completely confidential and not shared with other students<br><br>Student writing responds to structured instructor questions

a	b	c	d	e	f	g	h	i
Strongly Prefer Left		Somewhat Prefer Left		Indifferent		Somewhat Prefer Right		Strongly Prefer Right
